# Supplementary material for: Marine-Derived Actinomycetes: Biodegradation of Plastics and Formation of PHA Bioplastics—A Circular Bioeconomy Approach
Source: Mar Drugs. 2022 Dec 1;20(12):760. doi: 10.3390/md20120760 (PMC9783806; doi:10.3390/md20120760)
Supplement: Supplementary file 1 [file marinedrugs-20-00760-s001.zip › marinedrugs-1998342-supplementary.pdf]

Supplementary Materials

Marine-Derived Actinomycetes: Biodegradation of Plastics and Formation of PHA Bioplastics—A Circular Bioeconomy Approach

Juliana Oliveira [1,2], Pedro L. Almeida [3,4], Rita G. Sobral [1,2], Nídia D. Lourenço [1,2] and Susana P. Gaudêncio [1,2,*]

[1] Associate Laboratory i4HB–Institute for Health and Bioeconomy, NOVA School of Science and Technology, NOVA University Lisbon, 2819-516 Caparica, Portugal
[2] UCIBIO-Applied Molecular Biosciences Unit, Chemistry and Life Science Departments, NOVA School of Science and Technology, NOVA University of Lisbon, 2819-516 Caparica, Portugal
[3] I3N–CENIMAT, Materials Science Department, School of Science and Technology, NOVA University Lisbon, 2819-516 Caparica, Portugal
[4] Physics Department, Instituto Superior de Engenharia de Lisboa, Instituto Politécnico de Lisboa, 1959-007 Lisbon, Portugal
* Correspondence: s.gaudencio@fct.unl.pt; Tel.: +351-21-2948300; Fax: +351-21-2948550

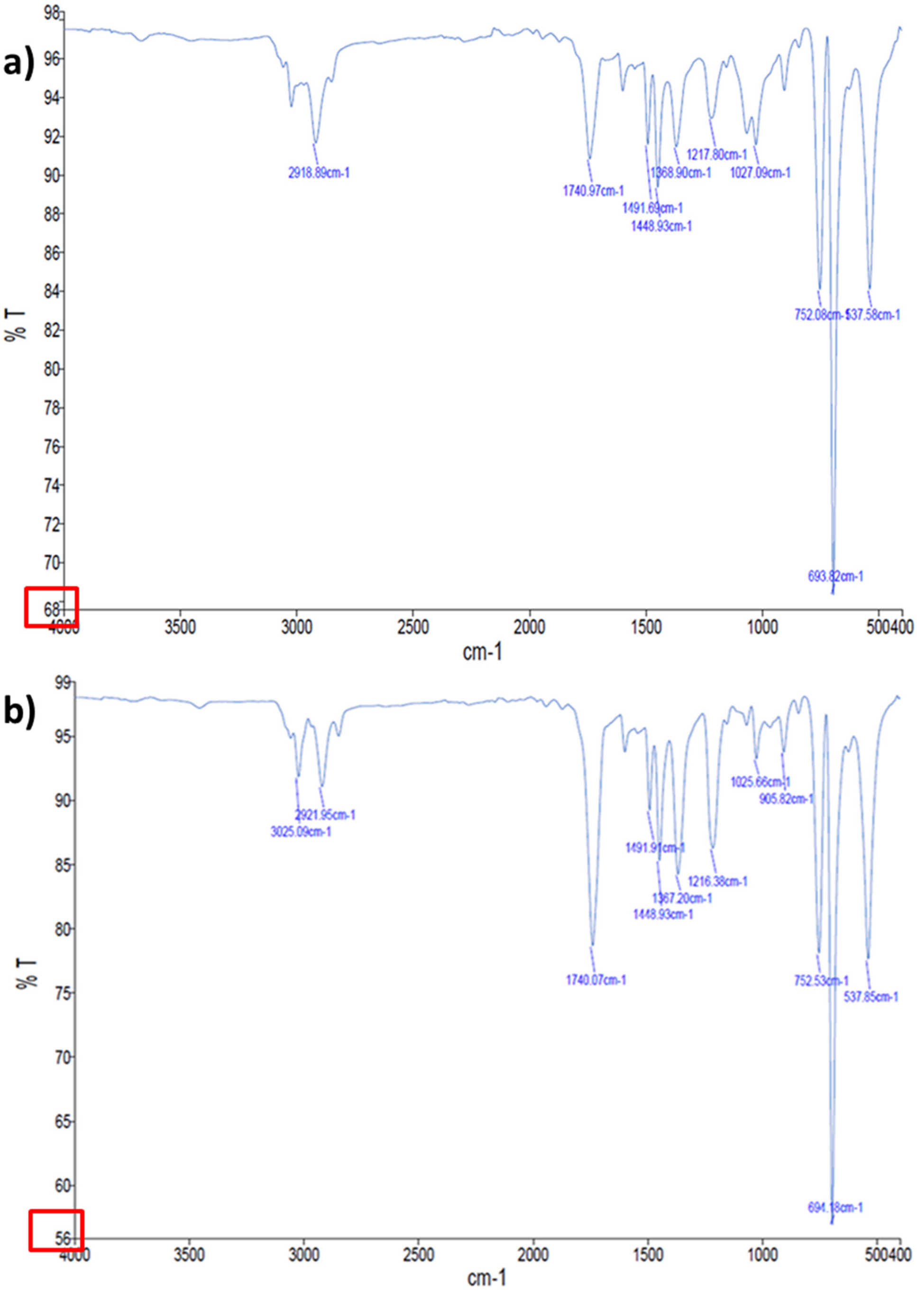

Figure S1. FTIR-ATR spectra of PS thin films incubated with *S. gougerotti*: a) in culture media without yeast extract; b) in culture media supplemented with yeast extract. Red rectangle highlights the increase in the intensity of the bands with the yeast extract conditions, which corresponds to polymer oxidation and a decrease in PS chain polymerization.

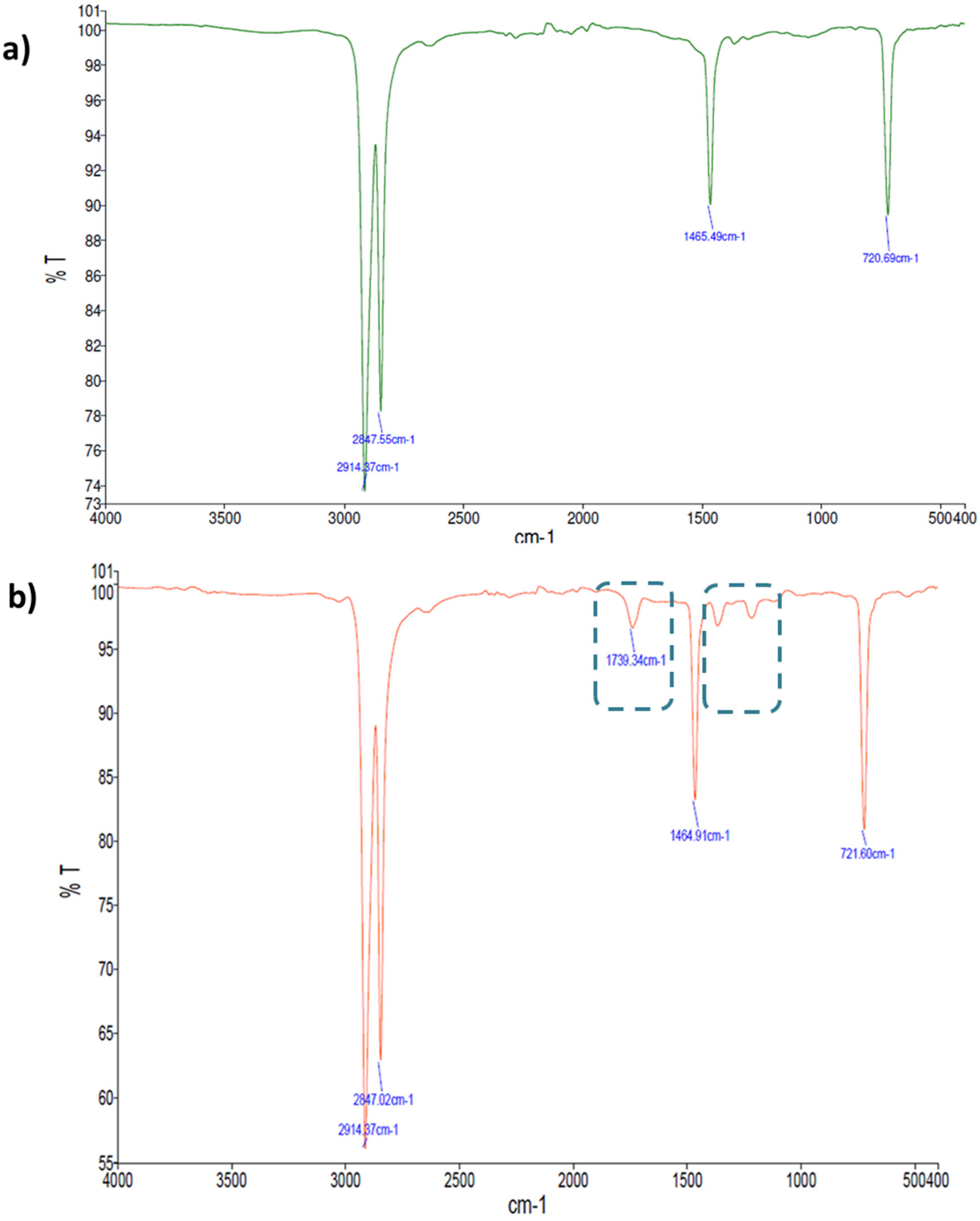

Figure S2. FTIR-ATR spectra of LDPE thin films incubated with *M. matsumotoense*. a) LPDE thin film control; b) LDPE thin film incubated with *M. matsumotoense*. The rectangles correspond to the new observed bands after incubation, at 1739 cm^{-1} and at 1367 and 1217 cm^{-1}, corresponding to carbonyl group formation and a decrease of LDPE chain polymerization, respectively.

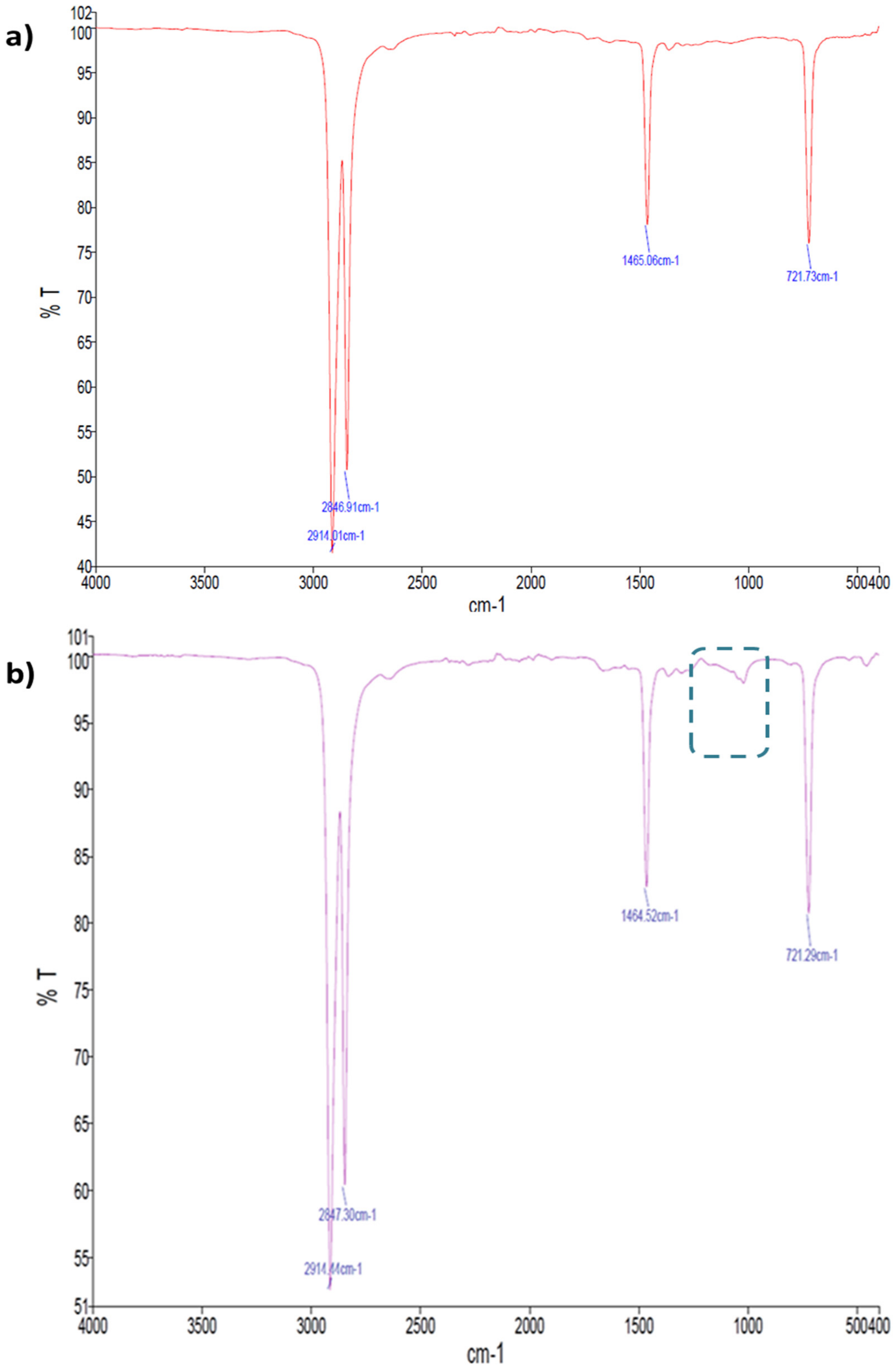

Figure S3. FTIR-ATR spectra of LDPE UV thin films incubated with *M. matsumotoense*. a) LPDE UV thin film control; b) LDPE UV thin film incubated with *M. matsumotoense*. The rectangle corresponds to the new observed bands after incubation, at 1096 cm^{-1}, indicating the presence of oxidized groups (groups containing -OH), which results from bacterial biodegradation.

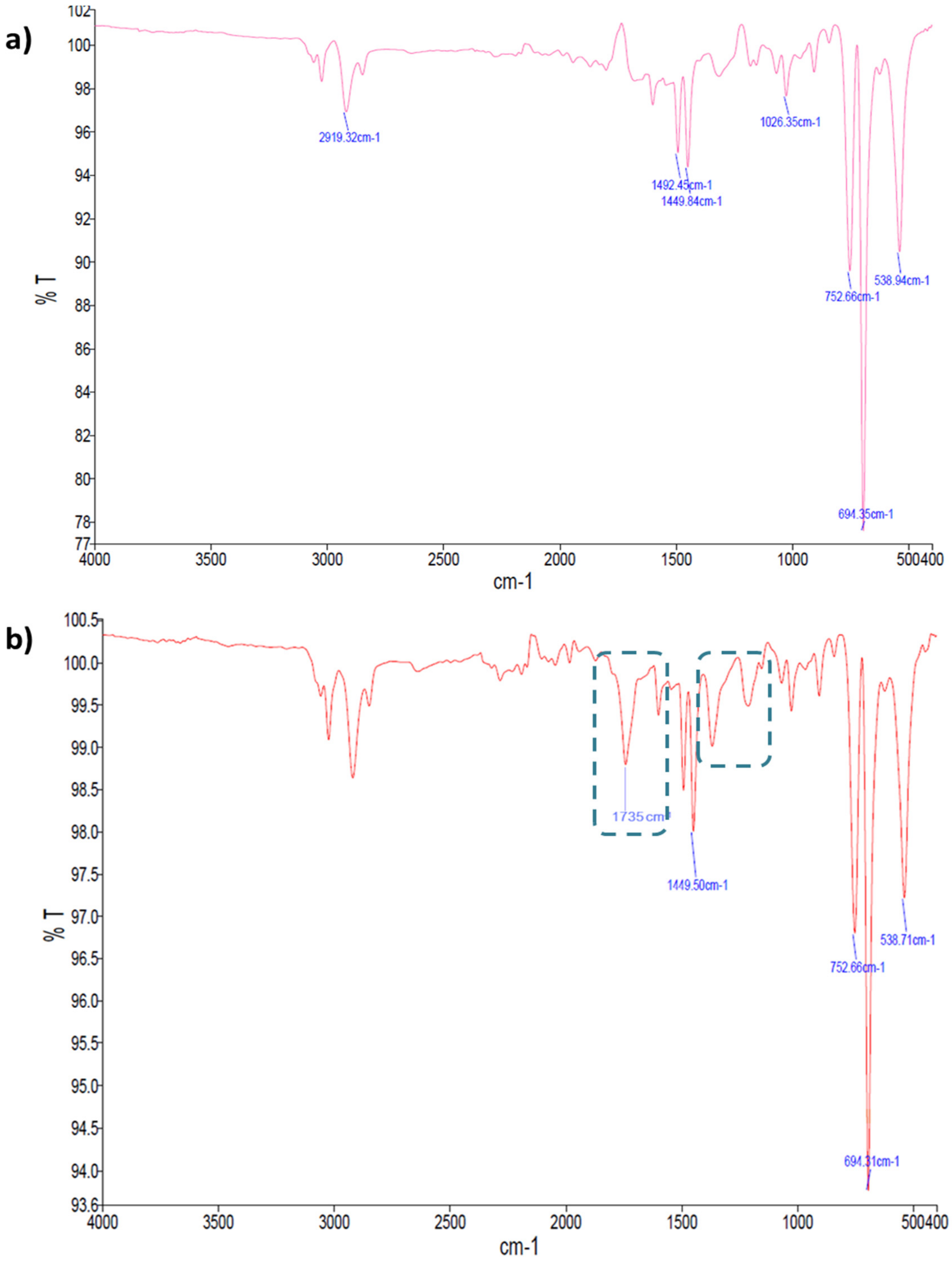

Figure S4. FTIR-ATR spectra of PS thin films incubated with *M. matsumotoense*. a) PS thin film control; b) PS thin film incubated with *M. matsumotoense*. The rectangle corresponds to the new observed bands after incubation, at 1735 cm^{-1} and at 1367 and 1217 cm^{-1}, corresponding to carbonyl group formation and a decrease in PS chain polymerization, respectively. This reveals biodegradation of the polymers by the secretion of enzymes from the microorganisms, which leads to polymeric disintegration.

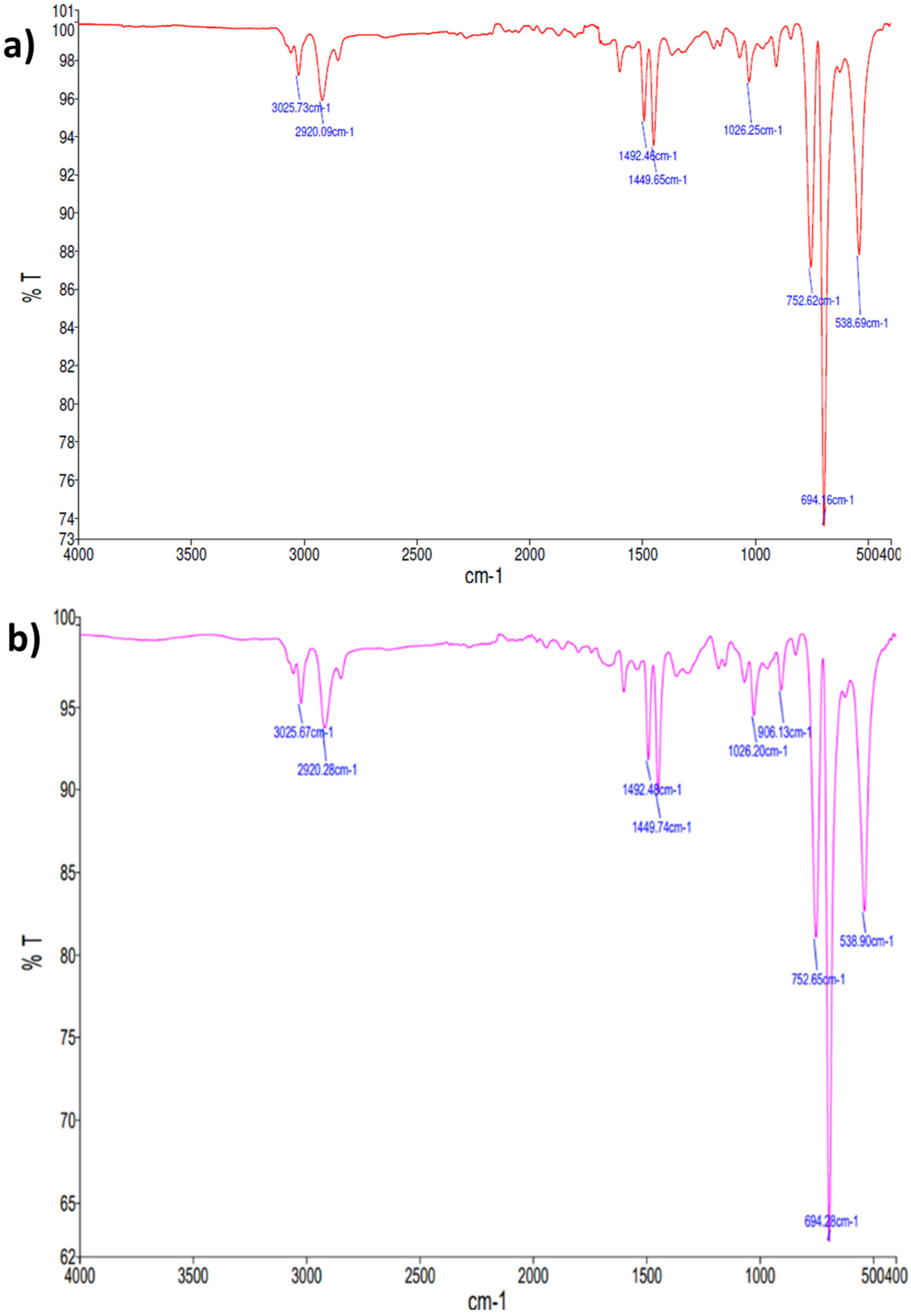

Figure S5. FTIR-ATR spectra of PS UV thin films incubated with *M. matsumotoense*. a) PS UV thin film control; b) PS UV thin film incubated with *M. matsumotoense*. An increase of the bands intensity after incubation with *M. matsumotoense* was observed, revealing chain oxidation and a decrease in PS UV chain polymerization.

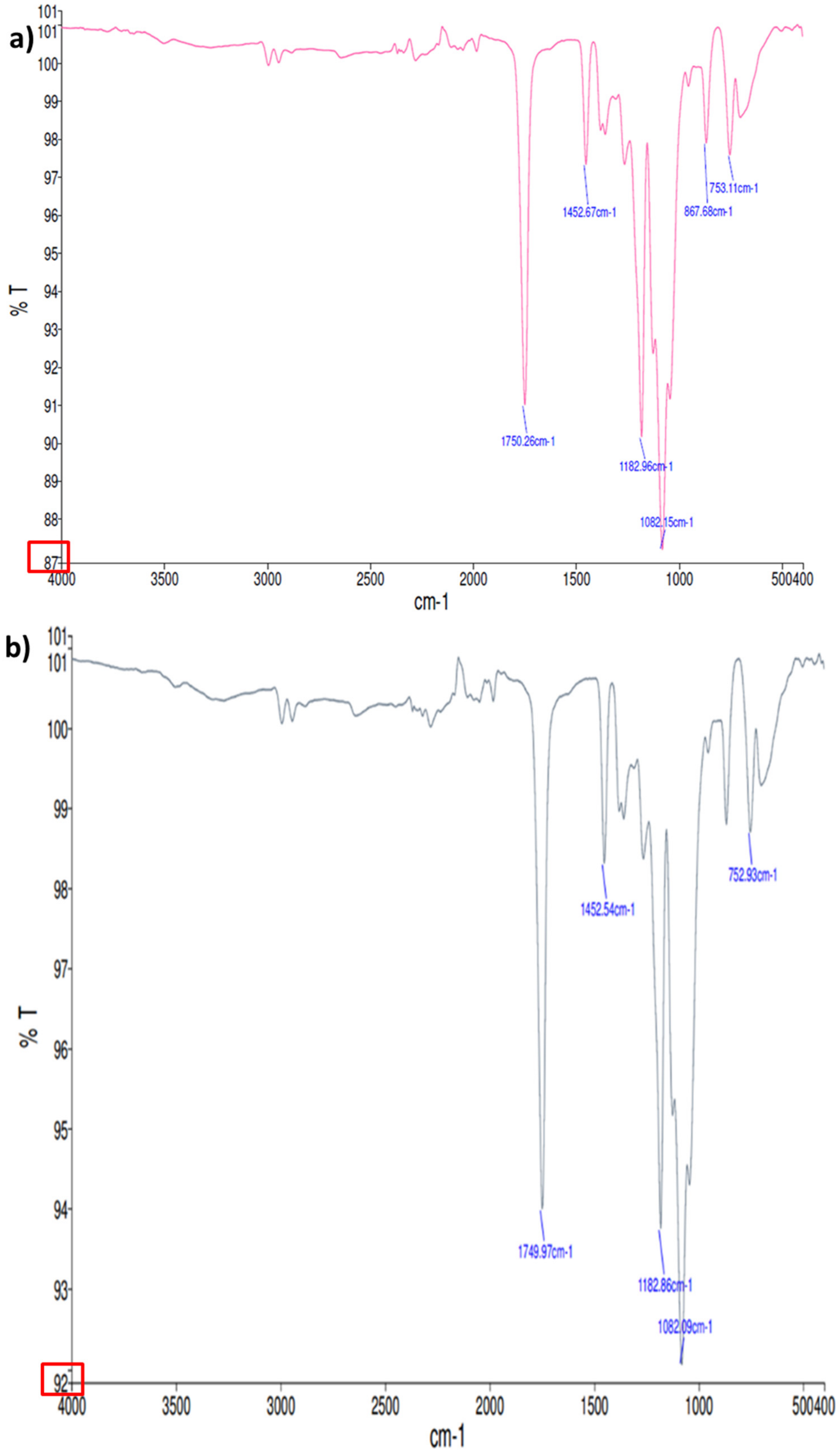

Figure S6. FTIR-ATR spectra of PLA thin films incubated with *N. prasina*. a) PLA thin film control; b) PLA thin film incubated with *N. prasina*. Red rectangle highlights the increase of the bands intensity after incubation with *N. prasina*, revealing chain oxidation and a decrease in PLA chain polymerization.

a)

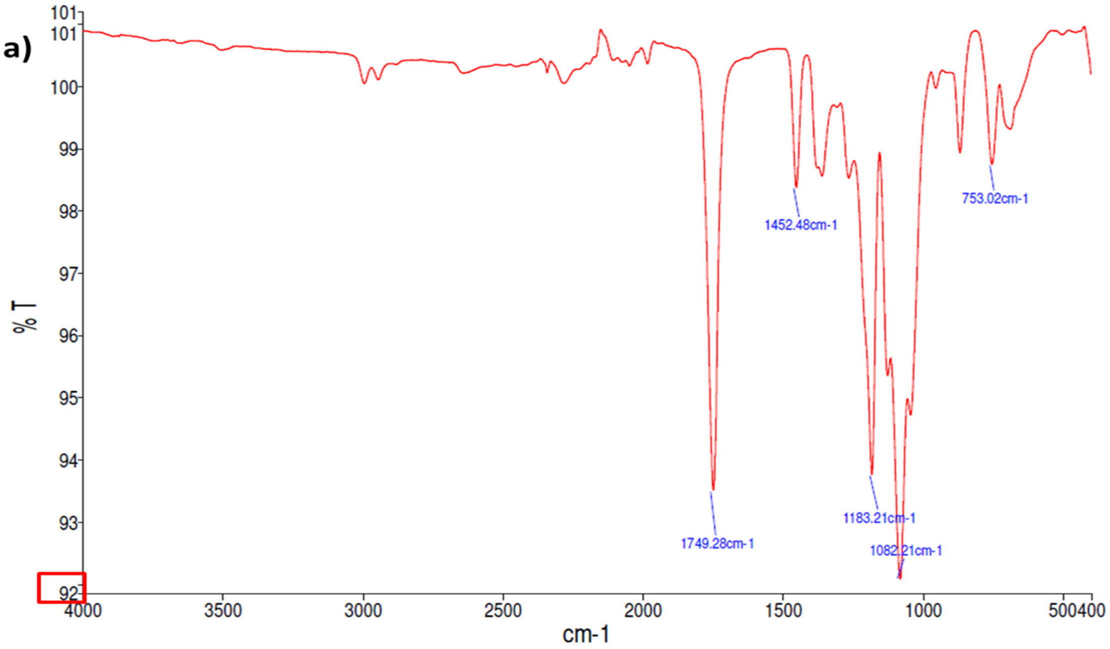
101
101
100
99
98
97
96
95
94
93
92
% T
1452.48cm-1
753.02cm-1
1749.28cm-1
1183.21cm-1
1082.21cm-1
4000
3500
3000
2500
2000
1500
1000
500400
cm-1

b)

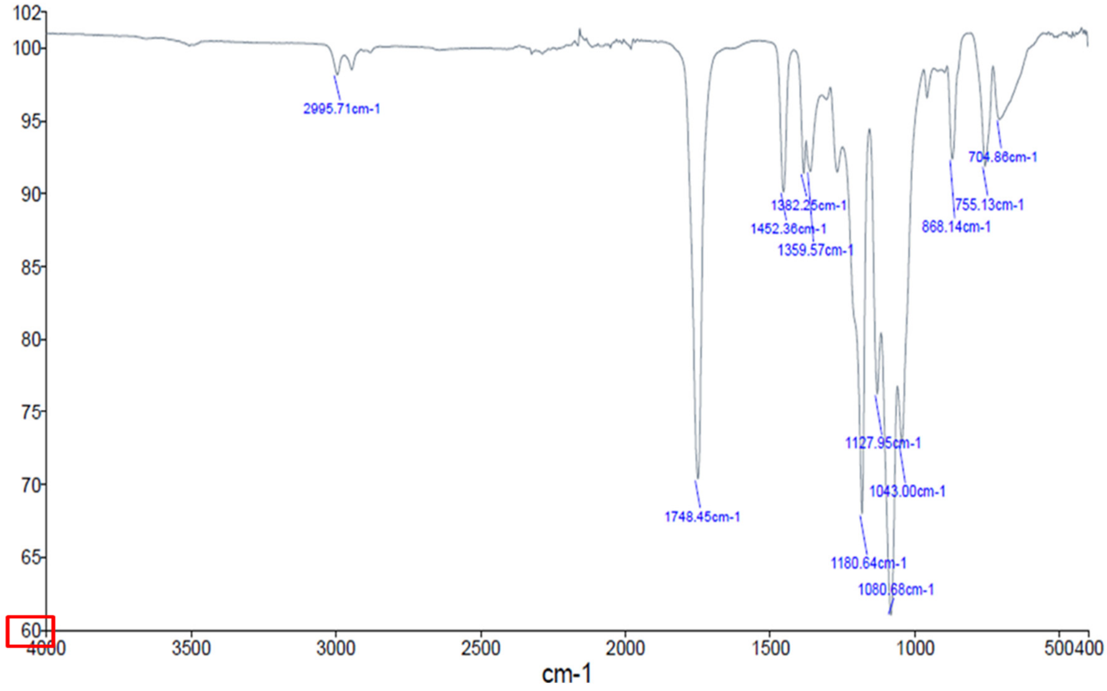
102
100
95
90
85
80
75
70
65
60
%T
2995.71cm-1
1382.25cm-1
1452.36cm-1
1359.57cm-1
704.86cm-1
755.13cm-1
868.14cm-1
1127.95cm-1
1043.00cm-1
1748.45cm-1
1180.64cm-1
1080.68cm-1
4000
3500
3000
2500
2000
1500
1000
500400
cm-1

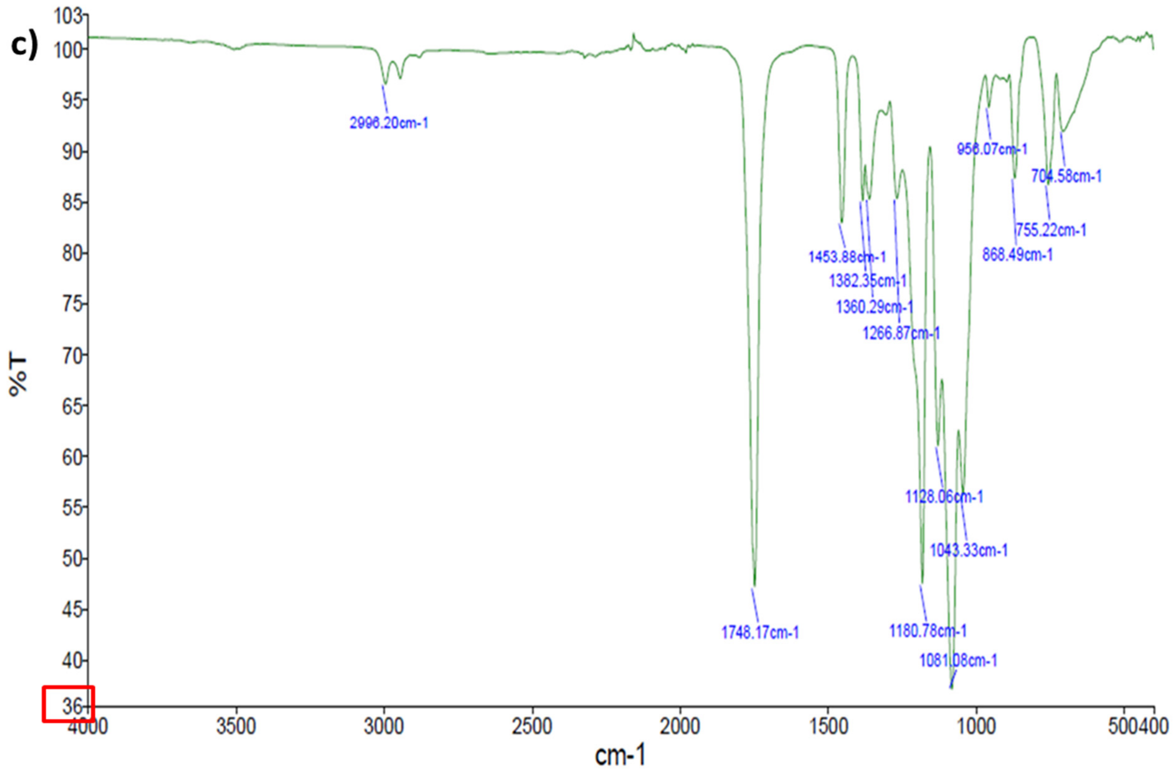

Figure S7. FTIR-ATR spectra of PLA UV thin films incubated with *N. prasina*. a) PLA UV thin film control; b) PLA UV thin film incubated with *N. prasina*; c) PLA UV thin film incubated with *N. prasina* in culture media supplemented with yeast extract. The red rectangles highlight the bands intensity differences with the different conditions, revealing higher oxidation. The bands at ~950 cm^{-1} and 700 cm^{-1} become more notorious and are characteristic of vinyl unsaturated groups and C-O double bounds, respectively. This is related to biodegradation.

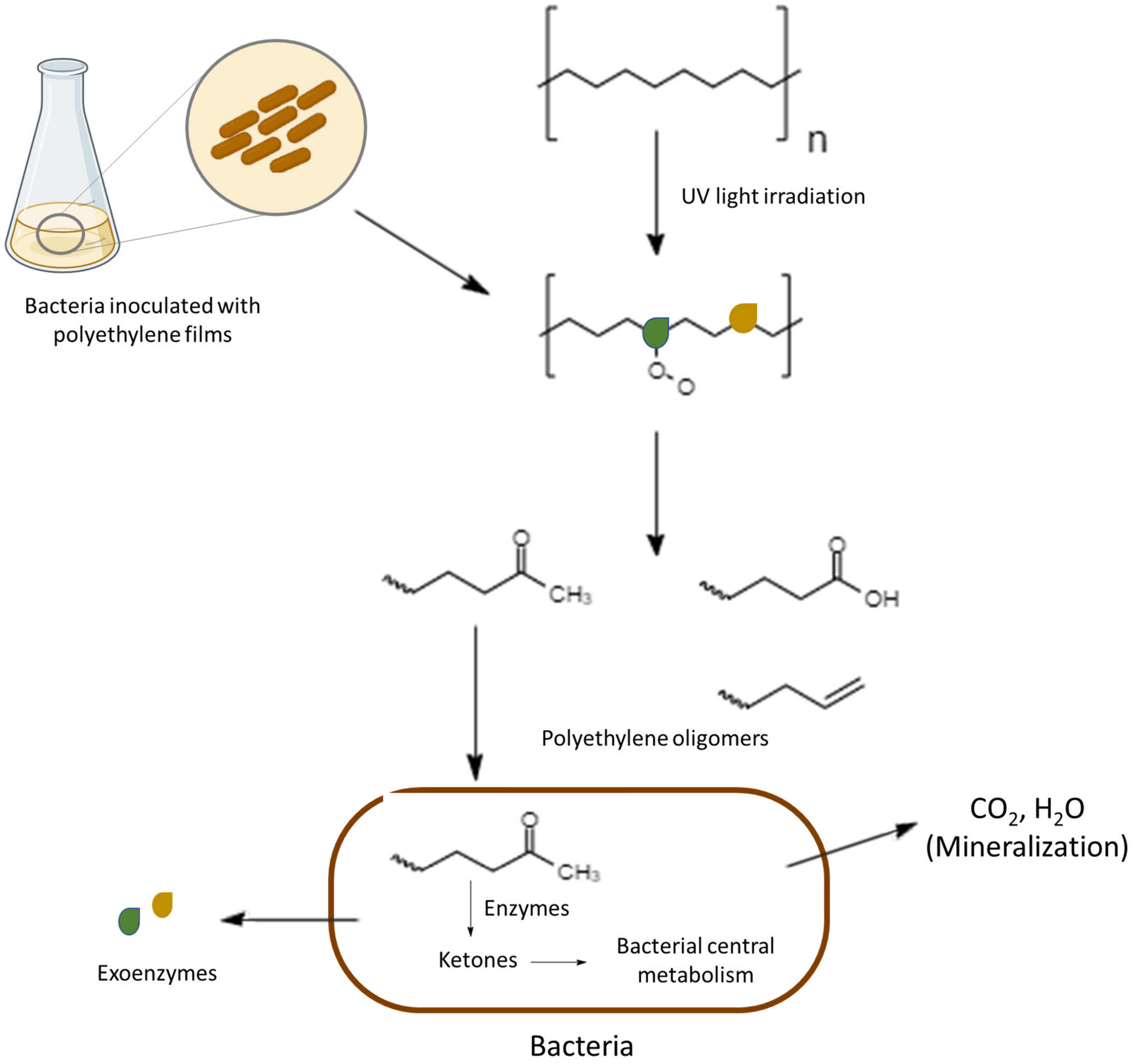

Figure S8. Mechanism of PE biodegradation by bacteria. During PE biodegradation carbonyl groups (-C=O) and oxidized groups such as moieties containing –OH groups are formed. These chemical changes were observed in the FTIR-ATR spectra. Adapted from Ghatge, S. et al. (2020). [1]

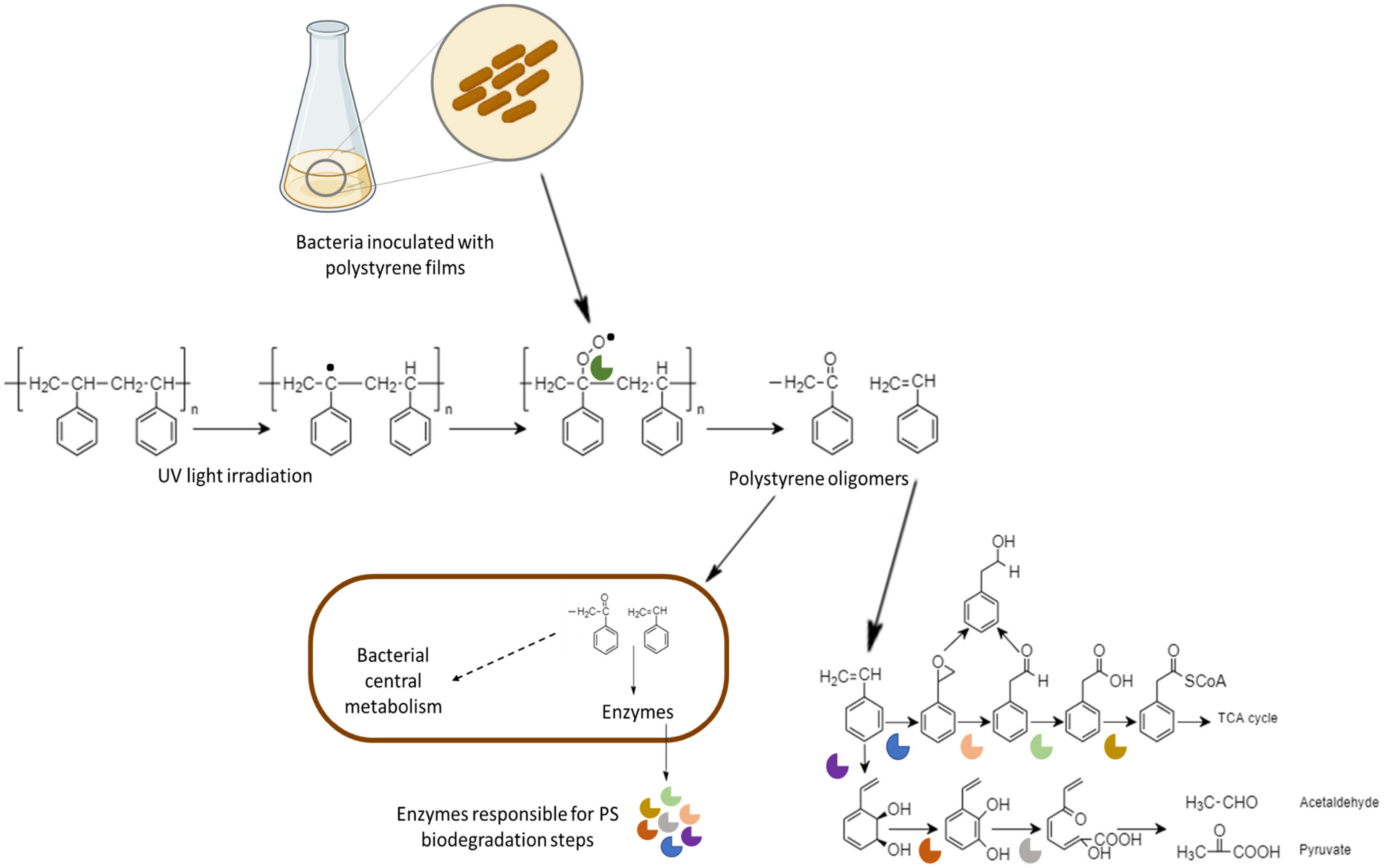

Figure S9. Mechanism for PS biodegradation by bacteria. During PS biodegradation several chemical groups are formed in the polymer surface, such as carbonyl groups (-C=O), alcohols (-C-OH), carboxylic acids (-COOH), esters (-COOR), and bound alterations in the benzene ring. These chemical changes were observed in the FTIR-ATR spectra. Adapted from Mooney, A. et al (2006). [2]

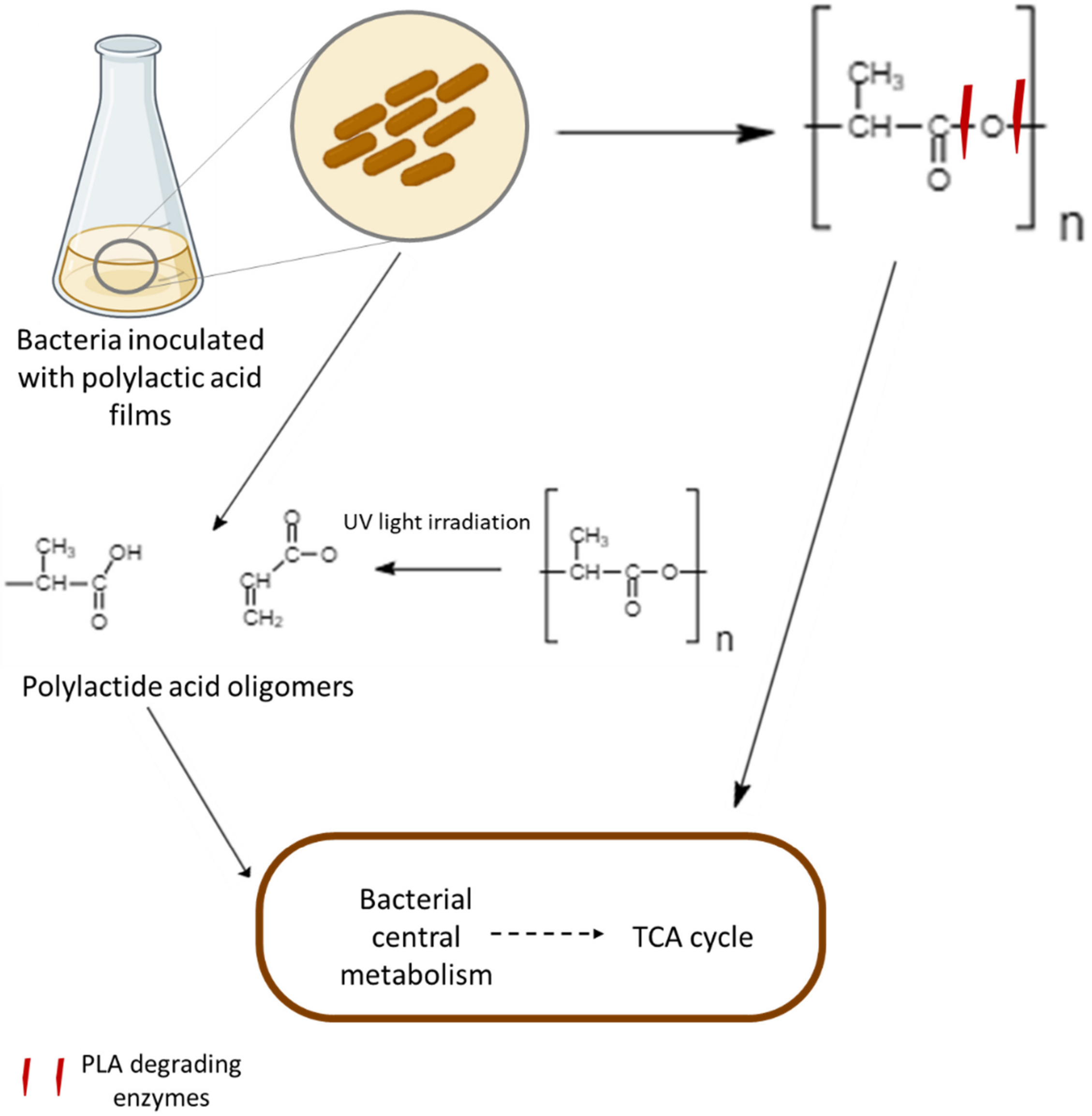

Figure S10. Mechanism for PLA acid biodegradation by bacteria. During PLA biodegradation chain oxidation and formation of vinyl unsaturated groups are observed. These chemical changes were observed in the FTIR-ATR spectra. Adapted from Pattanasuttichonlakul, W. et al. (2018). [3]

References:

[1] Ghatge, S.; Yang, Y.; Ahn, J.H.; Hur, H.-G. Biodegradation of polyethylene: a brief review. *Appl. Biol. Chem.* **2020**, *63*, doi:10.1186/s13765-020-00511-3.

[2] Mooney, A.; Ward, P.G.; O'Connor, K.E. Microbial degradation of styrene: biochemistry, molecular genetics, and perspectives for biotechnological applications. *Appl. Microbiol. Biotechnol.* **2006**, *72*, 1–10, doi:https://doi.org/10.1007/s00253-006-0443-1.

[3] Pattanasuttichonlakul, W.; Sombatsompop, N.; Prapagdee, B. Accelerating biodegradation of PLA using microbial consortium from dairy wastewater sludge combined with PLA-degrading bacterium. *Int. Biodeterior. Biodegradation* **2018**, *132*, 74–83, doi:https://doi.org/10.1016/j.ibiod.2018.05.014.
